# Association of Dental and Prosthetic Status with Oral Health-Related Quality of Life in Centenarians

**DOI:** 10.3390/ijerph182413219

**Published:** 2021-12-15

**Authors:** Caroline Sekundo, Eva Langowski, Samuel Kilian, Diana Wolff, Andreas Zenthöfer, Cornelia Frese

**Affiliations:** 1 Department of Conservative Dentistry, Clinic for Oral, Dental and Maxillofacial Diseases, University Hospital Heidelberg, 69120 Heidelberg, Germany; eva.langowski@med.uni-heidelberg.de (E.L.); diana.wolff@med.uni-heidelberg.de (D.W.); cornelia.frese@med.uni-heidelberg.de (C.F.); 2 Institute of Medical Biometry, University of Heidelberg, 69120 Heidelberg, Germany; kilian@imbi.uni-heidelberg.de; 3 Department of Prosthodontics, Clinic for Oral, Dental and Maxillofacial Diseases, University Hospital Heidelberg, 69120 Heidelberg, Germany; andreas.zenthoefer@med.uni-heidelberg.de

**Keywords:** centenarians, oral health, quality of life, dental prosthesis

## Abstract

To date, there is little evidence on centenarians’ dental and prosthetic status or their oral-health-related quality of life (OHRQoL). Therefore, the aim of this study was to assess possible associations between sociodemographic and oral health factors, including prosthetic needs in this special age group and their potential influence on OHRQoL. Persons born before 1920 were recruited from population registries in south-western Germany. Fifty-five centenarians participated and underwent a comprehensive oral examination. Cognitive capacity was evaluated using the short Mini-Mental State Examination (S-MMSE, max. 21 points). At an S-MMSE > 10, an analysis of OHRQoL by means of the Geriatric Oral Health Assessment Index (max. ADD-GOHAI score 60 points) was performed (n = 43). Bivariate statistics and a linear regression model were used after variable selection to analyze data. Centenarians presented with a mean (SD) of 22 (7.2) missing teeth. Complete (65.5%) or partial dentures (21.8%) in at least one jaw were most common. One-third of the dentures needed repair/replacement; 16% of the centenarians presented with denture sores. In 60% of cases, OHRQoL was rated unsatisfactory (ADD-GOHAI < 57). Trouble biting or chewing resulted in the lowest levels of OHRQoL. Fewer remaining teeth, reduced functional capacity and removable prostheses correlated with an impaired OHRQoL (r_s_ = −0.36, *p* = 0.01; r_s_ = −0.34, *p* = 0.01; r_s_ = −0.29, *p* = 0.03, respectively). After variable selection, the final linear regression model included only the number of missing teeth, the associated ADD-GOHAI score decreasing by 0.3 points per missing tooth. In conclusion, tooth loss and removable prostheses in need of repair or replacement are highly prevalent in centenarians. These factors seem to modulate OHRQoL negatively, assumedly due to impaired chewing function. Larger confirmatory studies are needed to validate these first results.

## 1. Introduction

Better health care and hygiene, reduced child mortality, sufficient food and improved medical care have led to longer lives [1]. As a result of these improved life expectancies in industrialized countries [2], the age range of so-called “seniors” encompasses many decades, creating new challenges for medical and dental care.

In the past, oral health care strategies in older people were mainly tailored with a focus on complete dentures, due to substantial tooth loss in this age group. However, the rate of edentulism has been declining in many countries [3], and other aspects of dental care have gained crucial importance, such as periodontology and restorative dentistry. Demographic changes are working against this downward trend. Although projections differ, some institutes prognosticate that prosthetic needs will be, consequently, increased in the future [4], while others forecast that some aspects of prosthetic treatment will disappear [5]. The realities, as far as they are currently known, suggest that prosthetic treatment needs will continue to play an important role in the aging population. However, although today’s younger population is expected to reach the high age of 100 years or more [6], there is little knowledge about prosthetic needs among the increasing group of the very old, i.e., centenarians.

Moreover, individuals with such prolonged lifespans also have an increased risk of age-related morbidity, and their quality of life may not be stable [7], including their oral-health-related quality of life (OHRQoL) [8]. When considering studies among “younger” older people, it has been shown that particularly those institutionalized and those with impaired cognitive function suffer from a low OHRQoL [9,10]. Whether or not this is equally valid for centenarians remains unclear [11].

To date, no study has assessed clinical data on centenarians’ dental and prosthetic status in a representative study sample and/or assessed oral-health-related quality of life. It is for this reason that the present study aims to assess the possible associations between sociodemographic and oral health factors as well as prosthetic needs in this special age group and how they might affect centenarians’ OHRQoL.

## 2. Materials and Methods

The Heidelberg Dental Centenarian Study (HD-100Z) was a cross-sectional survey and subsequent oral clinical examination among centenarians living in south-western Germany. The study was approved by the local review board of the Medical Faculty of the University of Heidelberg (S-168/2019) and registered with the German Clinical Trials Register (DRKS 00017128, date of registration: 20 May 2019). In view of sample sizes reached by previous studies on centenarians [12,13,14], the recruitment target was set at 50 participants. Informed written consent was obtained from all study participants, as well as their legal guardians if applicable. This study followed the Strengthening the Reporting of Observational Studies in Epidemiology (STROBE) guidelines [15].

Contact details of all centenarian citizens were provided by the 183 registries in the catchment area (from Karlsruhe in the south to Darmstadt in the north, and from the Rhine-Palatinate district in the east to the Neckar-Odenwald district in the west). Four-hundred-and-seventy-seven persons born before 1920 were registered and invited for study participation with no initial exclusion criteria. Fifty-five individuals were deceased or no longer living under the registered address at the time of contact (quality-neutral non-response = 11.5%). Among 422 valid contacts, 117 refused participation, and no contact could be established in 250 cases. Consequently, 55 centenarians participated and were visited at their residence (private home or elder care facility) between May and October 2019. Twelve centenarians had to be excluded from the evaluation of oral-health-related quality of life due to severely impaired cognitive capacity (for details on the exclusion criteria regarding cognitive capacity, please see Section 2.1 below).

### 2.1. Assessment of Cognitive Capacity and Sociodemographic Characteristics

A shortened version of the Mini-Mental State Examination (S-MMSE, max. 21 points) [16] was performed at the beginning of each visit. Tasks that may be biased by the impaired sensory functioning highly prevalent in this age group were not included [17]; this screening has proved itself useful in previous centenarian studies [18,19]. A score below 5 was considered a criterion for termination. At a score between 5 and 10, the clinical examination was performed, but no interview took place. However, objective information on sociodemographic data, such as officially recognized care levels or degrees of disability, etc., was registered, if available, by primary contacts or nursing staff. Sociodemographic items were assessed using the standardized sociodemographic survey for older adults aged 75–100 years used in the Fifth German Oral Health Study (DMS V) [20], including the assessment of educational levels, which were based upon participants’ highest obtained academic degree (low = no degree obtained or basic track, medium = intermediate track, high = academic track). The nursing-care level and disability were defined in accordance with German legislation [21,22,23].

### 2.2. Assessment of OHRQoL

OHRQoL was rated using the German version of the Geriatric Oral Health Assessment Index (GOHAI) [24]. The index includes 12 questions; each question is scored on a closed-option 5-point Likert scale (always–often–sometimes–seldom–never). Item 3,5 and 7 have to be inverted. The maximum additive score (ADD-GOHAI) is 60 points, equivalent to high OHRQoL. Results were also grouped based on previous studies [25,26,27] (≥57 satisfactory OHRQoL, 51–56 moderate OHRQoL, ≤50 severely impaired OHRQoL).

### 2.3. Clinical Examination

Each participant underwent a comprehensive oral examination, including assessment of the oral mucosa, their dental and prosthetic status. The dentition status, including caries experience, according to the DMF-T Index, was recorded according to WHO basic methods [28]. The main type of prosthesis worn was categorized according to Kerschbaum [29]: (1) full dental arch; (2) teeth partially missing without dental prosthesis; (3) crown(s); (4) fixed dental prosthesis (FDP); (5) removable dental prosthesis (RDP); (6) complete denture (CD). In the case of removable dentures, these were evaluated regarding their fit and potential necessary repairs, as well as their cleanliness, by two dentists, and a consensus decision was reached. Subsequently, dental functional capacity was evaluated as described by Nitschke et al. [30,31] and grouped into four resilience levels (1 = high functional capacity, 4 = low functional capacity). This measure has successfully been implemented in the Fifth German Oral Health Study (DMS V). [32] For this purpose, criteria regarding the ability to treat, oral hygiene and the person’s autonomy were evaluated using predefined fictitious scenarios. Treatment ability was defined as the centenarian’s ability to participate in dental treatment procedures. Oral hygiene describes the ability to attend a dental prophylaxis session and understand and implement oral-hygiene instructions. Autonomy describes the ability to choose to call a dentist and organize the visit.

For further information regarding interview and clinical examination methods, dental health behaviors, caries experience, functional capacity, periodontal and peri-implant conditions, please see our previous publications [33,34].

### 2.4. Statistical Analysis

Data analysis was performed with SPSS version 24.0 [35] and R version 4.0.3 [36]. Characteristics of the study population were presented by means of descriptive statistics. Means (SD) of continuous variables and proportion and frequency of categories of factor variables are reported. Spearman’s rank correlation coefficients were calculated to assess monotonous relationships of the GOHAI and clinical parameters.

In a second step, a linear regression after stepwise variable selection was performed to assess the influence of clinical and sociodemographic parameters on the additive GOHAI score (ADD-GOHAI). The variables were selected by AIC, and the full set of considered variables consisted of gender, level of education (low vs. medium/high), presence of disability, need of care, type of residence (home vs. institution), MMSE, the number of remaining teeth, the presence of removable prostheses and the functional capacity (RL 1 + 2 = low functional capacity vs. RL 3 + 4 = high functional capacity). *p* values are purely descriptive and regarded considerable if ≤0.05 due to the explorative nature of the study.

## 3. Results

### 3.1. Participants’ Characteristics and Dental/Prosthetic Status

The characteristics of the study population are summarized in Table 1. The mean age of the participants was 101.2 (SD = 1.6) years; most were female (83.6%). A mean of 22 (SD = 7.2) teeth were missing. All but three centenarians had had teeth replaced by prosthetic means; two of the exceptions had many remaining natural teeth but had edentulous spaces in their dental arches (category two according to Kerschbaum [29]). One centenarian was edentulous but could no longer wear her complete dentures; she was nevertheless categorized as cat. six, as this corresponded to her dental status. Although a full denture in at least one jaw was most frequently found (65.5%), only 36.4% of centenarians were completely edentulous. Partial dentures were the second most common type of prosthesis (21.8%). In total, 87.3% of centenarians presented with removable dentures, whereas 12.7% of centenarians had no need for removable prostheses and were either without prosthetic treatment or had fixed dental prostheses.

**Figure 1:**
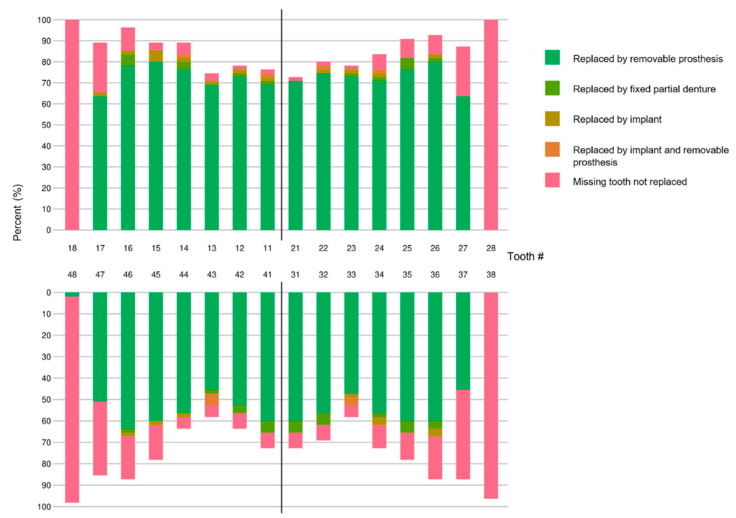
Prosthetically replaced teeth in the centenarian population (*n* = 55) depicted according to the FDI notation.

Regarding the localization, missing teeth in the maxilla were most frequently replaced by complete dentures (58.2%), followed by partial dentures (20.0%; cast partial dentures = 10.9%, combined dentures (telescope, interlock, attachment, etc.) = 9.1%). In the mandible, only 38.2% had complete dentures, equally followed by partial dentures (23.6%; cast partial dentures = 14.5%, combined dentures = 9.1%). In the mandible, 29.1% had no removable prosthesis. Figure 1 shows an overview of all replaced teeth and their means of replacement.

Considering the condition of removable dentures, only about two-thirds were sufficient; the others should have to be lined, repaired or even remade (Table 2). These limitations to the fit of many removable dentures were also evident in the examination of the oral mucosa. Inflammation caused by dentures (pressure points, denture-induced stomatitis) was the most frequent mucosal abnormality (present in 16% of participants, *n* = 9, i.e., 19% of denture wearers), followed by candida infection (2%, *n* = 1), leukoplakia (2%, *n* = 1), erythroplakia (2%, *n* = 1) and others (6%, *n* = 3). A total of 78% of centenarians showed no mucosal abnormalities. The majority of centenarians also showed deficits in their denture hygiene (Table 2).

### 3.2. Oral-Health-Related Quality of Life

The centenarians presented with a mean ADD-GOHAI score of 53.3 (SD = 6.7). According to the ADD-GOHAI scores, almost 40% of centenarians (*n* = 17) reported a satisfactory OHRQoL, 37% (*n* = 16) reported a moderate OHRQoL and 23% (*n* = 10) reported a severely impaired OHRQoL. Most reported no negative psychosocial impacts, discomfort or pain due to their oral status. However, trouble biting or chewing received the lowest OHRQoL scores, followed by limitations on the choice of food (Table 3).

### 3.3. Association between Centenarians’ Dental Functional Capacity, Dental/Prosthetic Status and OHRQoL

**Figure 2:**
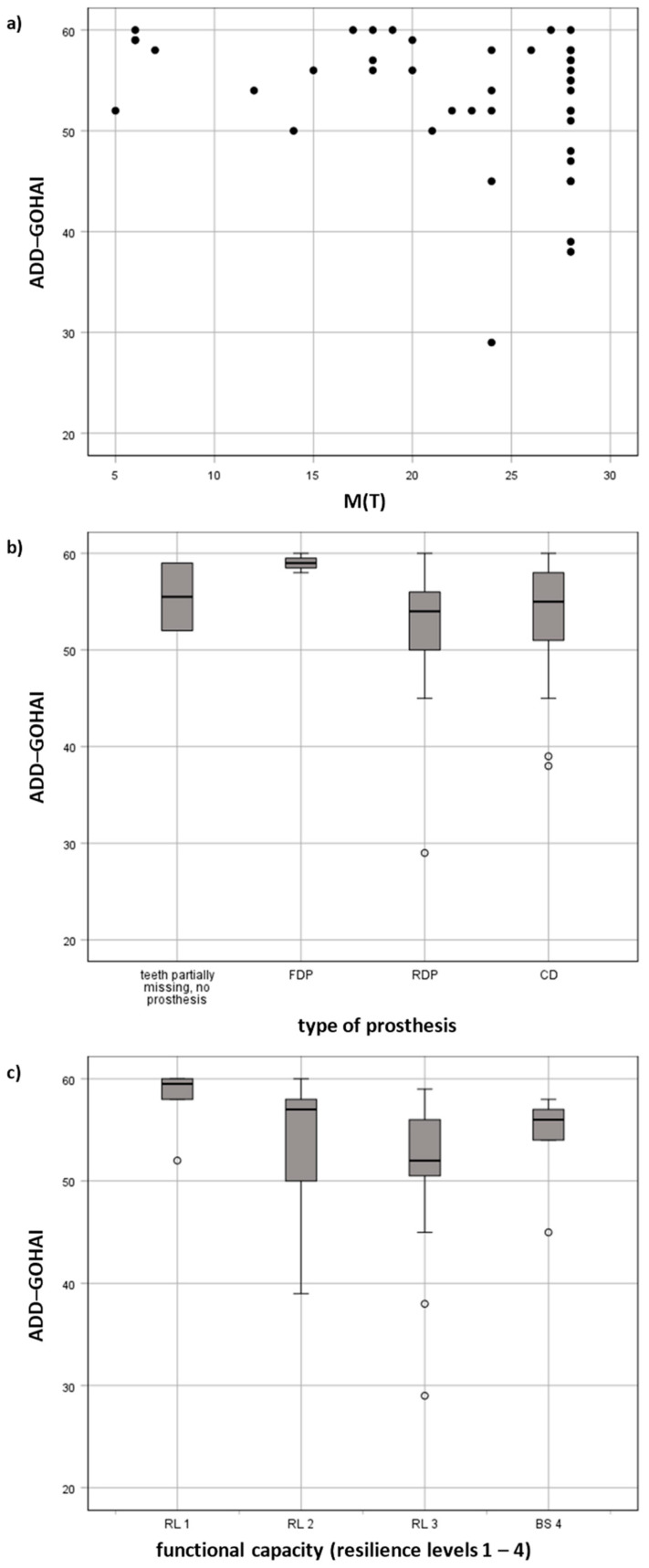
Influencing factors of OHRQoL in centenarians: (**a**) association of missing teeth M(T) with the ADD-GOHAI (scatter plot), (**b**) association of the type of dental prosthesis and (**c**) dental functional capacity with the ADD-GOHAI (box plots).

Among centenarians without removable prostheses, the mean ADD-GOHAI score was 57.6 (SD = 3.2); among those with removable prostheses, it fell to 52.8 (SD = 6.9). A lower ADD-GOHAI score correlated with fewer remaining teeth, reduced dental functional capacity and removable prostheses (Figure 2; r_s_ = −0.36, *p* = 0.01; r_s_ = −0.34, *p* = 0.01; r_s_ = −0.29, *p* = 0.03, respectively). Gender, level of education, disability, care levels, type of residence or the S-MMSE had no considerable impact on OHRQoL (*p* > 0.05). For details, please see Supplementary Table S1.

The results of the linear regression model are shown in Table 4. After variable selection, the final model only included the number of missing teeth (Figure 2a). The centenarians’ associated ADD-GOHAI scores decreased by 0.3 points per missing tooth.

## 4. Discussion

In this study—the first, worldwide, to assess dental care amongst centenarians in a clinical study—we show that, even at this exceptional age, the participants’ dental and prosthetic statuses varied widely. Although only a third of centenarians were completely edentulous, with a mean of 22 missing teeth, tooth loss was highly prevalent. The majority relied on removable prostheses, which were insufficiently fitting in approximately one-third of cases, highlighting the extent of prosthetic treatment needs. Many dentures required repair, underlining, exchange or, at the very least, cleaning.

This may explain why OHRQoL was mostly impaired due to trouble biting and chewing and was found not satisfactory in 60% of cases. The results of a systematic review [37] established a clear relationship between tooth loss and OHRQoL. This could be confirmed in the present study, where the total score of the GOHAI correlated with the number of missing teeth. With an increasing number of missing teeth, the oral-health-related quality of life decreased, as also shown in our linear regression model. Although prosthetic rehabilitation can compensate for the reduction in masticatory function that results from tooth loss to a certain extent, this effect could also be seen when evaluating the main type of prosthesis, i.e., centenarians with fixed dental prostheses also had a higher OHRQoL. Moreover, regarding removable dentures, it has been shown that masticatory function is lower when using older dentures (often due to poor fitting) [38,39,40]. This is thus a clear indication that maintaining teeth for as long as possible can have a positive effect on the quality of life and that the WHO goal of maintaining 20 or more teeth in older adults is a desirable endeavor. Identifying factors that are beneficial for maintaining one’s natural teeth should therefore be one of the main goals of further research. It must be mentioned, however, that trouble biting or chewing could also be partially age-related, as the hypoactivity of masticatory muscles during chewing may occur with increasing age [41].

In our study cohort, notably limited by its relatively small sample size, OHRQoL was not influenced by the question of institutionalization, nursing care or mental state, which are factors that have been shown to have an influence in studies with younger participants. We have previously shown that these factors also had no impact on centenarians’ cariological or periodontal status [33,34]. At the age of 100 years and older, all participants received some kind of help or nursing care, whether at home or in a care facility; this may have alleviated differences. In order to comprehensively assess their capabilities, frailty and cognition, the dental functional capacity according to Nitschke et al. [30,31] thus appears to be a valuable indicator. Resilience levels correlated significantly with centenarians’ OHRQoL.

Furthermore, the increased prevalence of denture-related mucosal lesions is remarkable. They were about four times more common in the group of centenarians than in younger German seniors, aged 75–100 years, examined in the Fifth German Oral Health Study (DMS V) [20]. It must be noted, however, that studies in other countries have found a similar or increased prevalence compared to our results, even among younger age groups [42,43,44]. Prosthesis-related changes to the oral mucosa are not necessarily associated with age [45]. Since older adults, especially centenarians, are often reduced in their mobility, access to dental care is limited [46]. Only approximately half take part in regular check-ups [34] and, due to their lower resilience, dentists may opt for uncomplicated, sometimes provisional care, possibly causing the relatively high prevalence of denture sores by these means.

This study has several limitations. Firstly, both its exploratory character and cross-sectional design do not allow for the determination of causal effects. In order to further explore the reasons for trouble biting and chewing, further studies assessing the masticatory function amongst different age groups with comparable prosthetic status are necessary. Secondly, the number of missing teeth in the linear regression model can only explain 8% of the variance of the ADD-GOHAI, underlining the necessity of further research into other cofactors. Factors to be explored in follow-up studies should include subjective general health assessments, social support items (which also encompass emotional, informational and affectionate support next to tangible support structures such as nursing care) and psychological wellbeing. Furthermore, the target population, living in an area in south Germany, may not be transferrable to all populations. Nonetheless, these results may show how demographic changes might affect dental care in industrialized countries. Moreover, the sample size is small; however, the achieved response rate of 13% is substantial considering the difficulties of contacting and motivating centenarians for clinical study participation.

## 5. Conclusions

In conclusion, tooth loss and the use of removable (insufficient) prostheses are highly prevalent in centenarians. This affects their OHRQoL, which was most frequently reduced due to trouble biting and chewing. Maintaining more natural teeth is the main beneficial factor correlating with a higher OHRQoL. Larger confirmatory studies are needed to validate these observations.

## Figures and Tables

**Table 1:** Characteristics of the study population.

Variable	Subcategory	Centenarians (*n* = 55)	
		Mean (SD)	*n* (%)
Age (years)		101.2 (1.6)	
Sex	Male		9 (16.4)
	Female		46 (83.6)
Level of education	Low		29 (52.7)
	Medium		13 (23.6)
	High		13 (23.6)
Recognized disability *	No		27 (49.1)
	Yes		28 (50.9)
Recognized nursing-care level *	No care level		7 (12.7)
	Slight, 1		3 (5.5)
	2		12 (21.8)
	3		20 (36.4)
	4		12 (21.8)
	Most severe, 5		1 (1.8)
Residence	Care facility		26 (47.3)
	At home		29 (52.7)
S-MMSE		15.0 (4.7)	
	5–10 points		12 (21.8)
	11–16 points		19 (34.5)
	17–21 points		24 (43.6)
M(T)		22.0 (7.2)	
Functional capacity according to Nitschke et al. [30,31]	Resilience level 1 (high functional capacity)		6 (10.9)
	Resilience level 2		13 (23.6)
	Resilience level 3		27 (49.1)
	Resilience level 4 (low functional capacity)		9 (16.4)
Main types of prosthesis according to Kerschbaum [29]	Full dental arch		0 (0.0)
	Teeth partially missing without dental prosthesis		2 (3.6)
	Crown(s)		0 (0.0)
	Fixed dental prosthesis		5 (9.1)
	Removable dental prosthesis		12 (21.8)
	Complete denture		36 (65.5)

* According to current German legislation [21,22,23].

**Table 2:** Prosthetic treatment needs of removable dentures.

Recommended Repair or Therapy	Maxilla *n* (%)	Mandible *n* (%)
No need for repair or treatment	30 (66.7)	26 (68.4)
Underlining necessary	5 (11.1)	5 (13.2)
Repair necessary	9 (20.0)	2 (5.3)
Treatment of denture-related mucosal lesion necessary	7 (14.6)	4 (8.3)
New dentures necessary	1 (2.2)	5 (13.2)
**Cleanliness**		
Good denture hygiene	18 (40.0)	15 (38.5)
Denture hygiene can be improved, acceptable	9 (20.0)	7 (17.9)
Denture hygiene acceptable, but professional laboratory cleaning recommended	8 (17.8)	8 (20.5)
Poor denture hygiene	10 (22.2)	9 (23.1)

**Table 3:** OHRQoL in centenarians: Geriatric Oral Health Assessment Index (*n* = 43).

Participants’ Answers *n* (%)						
Variable	Never	Seldom	Sometimes	Often	Always	Mean (SD)
**Physical function**						
1. Limitation of food choices	28 (65.12)	4 (9.30)	4 (9.30)	2 (4.65)	5 (11.63)	4.12 (1.42)
2. Trouble biting or chewing	17 (39.53)	1 (2.33)	7 (16.28)	7 (16.28)	11 (25.58)	**3.14 (1.68)**
3. Problems with swallowing comfortably	38 (88.37)	1 (2.33)	3 (6.98)	0	1 (2.33)	4.74 (0.79)
4. Problems with speaking clearly	36 (83.72)	0	2 (4.65)	4 (9.30)	1 (2.33)	4.53 (1.10)
						4.13 (0.87)
**Pain and discomfort**						
5. Discomfort when eating any kind of food	31 (72.09)	4 (9.30)	4 (9.30)	2 (4.65)	2 (4.65)	4.40 (1.14)
8. Used medication to relieve pain	37 (86.05)	4 (9.30)	1 (2.33)	0	1 (2.33)	4.77(0.72)
12. Sensitive to hot, cold or sweet foods	36 (83.72)	2 (4.65)	3 (6.98)	2 (4.65)	0	4.67 (0.81)
						4.60 (0.66)
**Psychosocial impacts**						
6. Limits contact with others	41 (95.35)	1 (2.33)	0	1 (2.33)	0	4.91 (0.48)
7. Pleased with look of teeth	1 (2.33)	2 (4.65)	8 (18.60)	7 (16.28)	25 (58.14)	4.23 (1.07)
9. Worried about teeth, gums or dentures	35 (81.40)	4 (9.30)	1 (2.33)	2 (4.65)	1 (2.33)	4.63 (0.93)
10. Self-conscious of teeth, gums or dentures	38 (88.37)	0	3 (6.98)	2 (4.65)	0	4.72 (0.80)
11. Uncomfortable eating in front of others	35 (81.40)	1 (2.33)	2 (4.65)	2 (4.65)	3 (6.98)	4.47 (1.22)
						4.59 (0.67)
ADD-GOHAI						53.33 (6.72)
SC-GOHAI						2.09 (2.14)

**Table 4:** Linear regression model to predict centenarians’ OHRQoL.

(Dependent Variable = ADD-GOHAI Score)				
Coefficients	Estimate B	*p*	95% CI for B	
			Lower Bound	Upper Bound
Intercept	59.61	<0.001	53.32	65.90
M(T)	−0.29	0.04	−0.56	−0.01

Adjusted R^2^ = 0.08.

## Data Availability

Data are available from the corresponding authors upon reasonable request.

## Supplementary Materials

The following are available online at https://www.mdpi.com/article/10.3390/ijerph182413219/s1, Table S1: Bivariate association of centenarians’ characteristics and the ADD-GOHAI score (Spearman’s rank correlation).
