# Supplementary material for: Association of Dental and Prosthetic Status with Oral Health-Related Quality of Life in Centenarians
Source: Int J Environ Res Public Health. 2021 Dec 15;18(24):13219. doi: 10.3390/ijerph182413219 (PMC8701196; doi:10.3390/ijerph182413219)
Supplement: Supplementary file 1 [file ijerph-18-13219-s001.zip › ijerph-1487403-supplementary.pdf]

Table S1. Bivariate association of centenarians' characteristics and the ADD-GOHAI score (Spearman's rank correlation)

Variable		ADD-GOHAI score	r_s	p
		Mean (SD)		
Sex	Male	54.25 (3.92)	0.02	0.45
	Female	53.11 (7.23)		
Level of education	Low	53.26 (6.13)	0.06	0.36
	Medium	53.00 (9.42)		
	High	53.73 (5.97)		
Recognized disability*	No	54.33 (4.56)	0.05	0.38
	Yes	52.36 (8.27)		
Recognized nursing care level*	No care level	56.14 (3.93)	- 0.08	0.31
	Slight 1	55.50 (4.95)		
	2	49.45 (10.56)		
	3	53.82 (4.82)		
	Severe 4	55.00 (2.83)		
Residence	Care facility	52.95 (5.84)	0.14	0.19
	At home	53.63 (7.45)		
S-MMSE	11-16 points	52.30 (7.01)	0.18	0.13
	17-21 points	54.46 (6.44)		
M(T)	0-14	56.00 (3.96)	**- 0.36**	**0.01**
	15-28	52.81 (7.05)		
Functional capacity according to Nitschke et al.[30, 31]	Resilience level 1 (high functional capacity)	58.17 (3.13)	**- 0.34**	**0.01**
	Resilience level 2	53.69 (6.66)		
	Resilience level 3	51.37 (7.40)		
	Resilience level 4 (low functional capacity)	54.00 (5.24)		
Removable prosthesis	No	57.60 (3.21)	**- 0.29**	**0.03**
	Yes	52.76 (6.88)		

* According to current German legislation [21-23]
